# Exploring functioning and health-related quality of life in patients referred to a diagnostic cancer pathway for non-specific serious symptoms

**DOI:** 10.1007/s00520-025-09825-8

**Published:** 2025-08-20

**Authors:** Jannie Rhod Bloch-Nielsen, Thomas Maribo, Helene Nørgaard Kristensen, Jaana Paltamaa, Anne Mette Schmidt

**Affiliations:** 1 Medical Diagnostic Centre, University Clinic for Innovative Patient Pathways, Regional Hospital Central Jutland, Silkeborg, Denmark; 2 Medical Diagnostic Centre, Department of Physiotherapy and Occupational Therapy, Silkeborg Regional Hospital, Falkevej 1-3, 8600 Silkeborg, Denmark; 3 Department of Public Health, Aarhus University, Aarhus, Denmark; 4 DEFACTUM, Central Denmark Region, Aarhus, Denmark; 5 The School of Health and Social Studies, Jamk University of Applied Sciences, Jyväskylä, Finland

**Keywords:** Functioning, Cancer, Quality of life, WHODAS, EQ5D

## Abstract

**Objective:**

To assess functioning and health-related quality of life (HRQoL) in patients referred to a cancer diagnostic pathway for non-specific serious symptoms, and to explore whether changes over 3 months differ among patients diagnosed with cancer, another serious diagnosis, or no serious diagnosis.

**Methods:**

A prospective cohort study was conducted at a hospital-based cancer diagnostic clinic. Functioning and HRQoL were assessed at baseline and after 3 months using the 36-item WHO Disability Assessment Schedule 2.0 (WHODAS 2.0) and the EuroQol 5-Domain 5-Level (EQ-5D-5L), respectively.

**Results:**

A total of 347 patients were included, with 242 completing follow-up. At baseline, patients with another serious diagnosis reported the highest functioning difficulties (median WHODAS: 25) and lowest HRQoL (median EQ-5D-5L: 0.705). Functioning scores improved modestly across all groups at 3 months, with no statistically significant differences between them. However, HRQoL improved significantly only in patients with another serious diagnosis, compared to those with cancer or no serious diagnosis (*p* = 0.04).

**Conclusion:**

Patients referred to a cancer diagnostic pathway for non-specific serious symptoms experience considerable functioning difficulties and reduced HRQoL at referral. While functioning improved modestly across all groups, only patients with another serious diagnosis showed a significant improvement in HRQoL. These findings highlight the need for early assessment and support for all patients in this pathway, irrespective of the final diagnosis, to ensure equitable care and timely rehabilitation when needed.

**Supplementary Information:**

The online version contains supplementary material available at 10.1007/s00520-025-09825-8.

## Introduction

The growing incidence of cancer, responsible for 10 million deaths and 19.3 million new cases annually, poses a substantial burden on global morbidity, mortality, and functioning[1–3]. Efforts to address this include the implementation of urgent organ-specific cancer pathways observed in several countries[4]. However, approximately half of cancer patients present with vague or non-specific serious symptoms such as weight loss, fatigue, pain, loss of appetite, and reduced physical ability[4, 5]. Consequently, diagnostic cancer pathways for patients with serious non-specific symptoms have been established[4]. Almost a quarter of these patients had significant comorbidities before referral to this pathway, and comorbidity is a known risk factor for disability[6–8]. Cancer prevalence among patients referred to cancer pathways for serious non-specific symptoms ranges from 11 to 35%[4]. Notably, 35–64% of these patients receive another serious diagnosis, either malignant or non-malignant with rheumatological, musculoskeletal, and gastrointestinal disorders being the most common[4, 9, 10]. Thus, patients referred to cancer pathways for serious non-specific symptoms may experience functioning difficulties and reduced quality of life. Studies have shown that cancer survivors have many different needs[11]. Patients referred to a cancer diagnostic pathway with serious non-specific symptoms but not diagnosed with cancer exhibit similar symptoms as those diagnosed with cancer[4]. Rehabilitation has been shown to effectively improve health, functioning, and quality of life in cancer survivors[12, 13]. Rehabilitation consists of interventions targeted at individuals with limitations in physical, mental, cognitive, and/or social functioning due to a health condition[14, 15]. The assessment of functioning and disability is integrated into the International Classification of Diseases, 11th Revision (ICD11) using the generic questionnaire World Health Organization Disability Assessment Schedule 2.0 (WHODAS 2.0)[16, 17].

In a previous explorative study, we found that 69% of patients diagnosed with cancer through this pathway were referred to rehabilitation following a needs assessment (submitted but not yet accepted for publication). Additionally, we found that patients without a cancer diagnosis appeared to face similar challenges but were not offered the same opportunity for a needs assessment. Patients referred to a cancer diagnostic pathway for non-specific serious symptoms represent a diagnostically complex and potentially vulnerable population [4]. Despite their symptom burden and high rate of serious diagnoses both malignant and non-malignant their functioning and HRQoL remain poorly described in the literature. Understanding functioning and HRQoL at the point of referral is crucial for identifying early rehabilitation needs, tailoring support, and ensuring equity in care regardless of diagnosis.

The aim of this study was therefore to describe functioning and HRQoL at the time of referral in patients presenting with non-specific serious symptoms of cancer. A secondary aim was to explore whether changes in functioning and HRQoL over a 3-month period differed among patients subsequently diagnosed with cancer, those diagnosed with another serious diagnosis, and those without a serious diagnosis.

## Methods

### Study design and setting

A cohort study with prospective data and a 3-month follow-up was conducted. Data was collected at the cancer diagnostic clinic at Silkeborg Regional Hospital, Denmark, serving a catchment area of approximately 242,000 residents. Annually, about 700 patients with non-specific, serious symptoms are referred to the clinic by general practitioners or other hospital departments. Patients undergo individual diagnostic programs based on their medical history and test results. The study followed the STROBE guidelines for observational research[18].

### Participants

All patients over the age of 18 referred to the cancer diagnostic pathway for serious non-specific symptoms between June 2023 and June 2024 were invited to participate. Patients were excluded if they were unable to read and understand Danish, had a severe psychiatric disorder, dementia, or other cognitive impairment preventing them from answering the questionnaires. An experienced secretary assessed these criteria, scheduling the patient’s first consultation. Patients received written information in their notice letter and provided informed consent online before answering the questionnaire.

### Data collection

Data was collected at referral (baseline) and after 3 months. At baseline, patients completed a self-administered questionnaire online at home before their consultation or arrived 30 min early to complete it on a tablet. In case of no response, a reminder was sent 1–3 days before the consultation.

Three-month self-administered follow-up questionnaires were completed online or, if needed, by telephone interview. In case of no response, two reminders were sent at 7-day intervals. The first author handled all reminders and interviews.

Research Electronic Data Capture (REDCap) was used to send questionnaires and collect data.

### Variables

Patient characteristics obtained at baseline included information about sex, age, cohabitant status, education, occupation, smoking and alcohol habits, and comorbidity. Cohabitant status was categorized into married/cohabiting, living alone, or other. Education was classified using the International Standard Classification of Education[19]. Occupation was categorized as employed/self-employed, unemployed, receiving sickness/activity compensation, retired, or other. Smoking was categorized as non-smoker, former smoker, and current smoker, and alcohol habits was grouped into two categories based on weekly intake ≤ 10 units/week or > 10 units/week. The Charlson Comorbidity Index was used to identify specific ICD-10 conditions up to 10 years before referral[20, 21]. Comorbidity was categorized into none (index score 0), moderate (index score 1 and 2), and high (index score 3 or more)[9]. At the 3-month follow-up, data was obtained on the three diagnostic groups: patients with a cancer diagnosis, those with another serious diagnosis, and those with no serious diagnosis. Data on mortality was collected from the patients’ electronic medical records.

Functioning was self-reported using the World Health Organization Disability Assessment Schedule 2.0 (WHODAS 2.0) 36-item, a standardized and validated generic questionnaire for assessing daily life functioning[22, 23]. The questionnaire has been developed based on the International Classification of Functioning, Disability, and Health (ICF). It comprises 36 items divided into six domains: Cognition, Mobility, Self-care, Getting along, Life activities, and Participation. It assess restrictions that the patient has experienced over the past 30 days[16]. The five answer options—mild, moderate, severe, and extreme limitations/cannot perform—are scored by 0–4 points. A total sum score of 0 to 144 is calculated using a simple method. The higher the score, the greater the functional difficulties[16]. Patients with incomplete WHODAS 2.0 assessments at baseline were excluded. Permission to use WHODAS 2.0 was granted by the WHO (reg. 391,960).

HRQoL was assessed using the EuroQol questionnaire (EQ-5D-5L), a validated generic instrument[24, 25]. The questionnaire assesses the patient’s current limitations across five domains: mobility, personal care, usual activities, pain/discomfort, and anxiety/depression. Responses are divided into five categories: no, easy, moderate, large, extreme problems/not possible to perform[26]. Based on a Danish set of values, HRQoL was calculated and given an index value between 1 (best possible health) and − 0.757 (worst possible health)[27]. Missing data were handled in accordance with the manual[26]. Permission to use the EQ-5D-5L was granted by EuroQol (reg. number 54499).

### Ethics

The study was conducted according to the Declaration of Helsinki[28]. The study was registered at The Danish Data Protection Agency (Journal number: 1–16-02–47-23). Informed patient consent was obtained before inclusion, and data was stored and anonymized according to the guidelines. According to Danish law, research using questionnaires does not require approval from the ethics committee (journal number: 1–10-72–109-23).

### Statistical methods

Descriptive statistics were used to summarize patient characteristics. Categorical variables were described as counts and proportions (%). Continuous variables were described with mean and standard division (SD) if normality was accepted, and otherwise as median and interquartile range (IQR).

Multiple regression was used to compare the change score (calculated as the difference between baseline and 3-month follow-up) between the three diagnostic groups in a crude analysis and an analysis with adjustment for covariates. Robust variance estimation was used to allow for possible non-normality and heteroscedasticity of residuals. The covariates included in the multivariate model were chosen a priori and consisted of sex, age, cohabitant status, education, occupation, smoking, alcohol habits, and comorbidity. All covariates were included in the adjusted models as categorical variables, except for age, which was treated as a continuous variable. No categories were collapsed. Dropout analysis was performed. Analyses were conducted using STATA18 statistical software.

## Results

### Participants

**Fig. 1:**
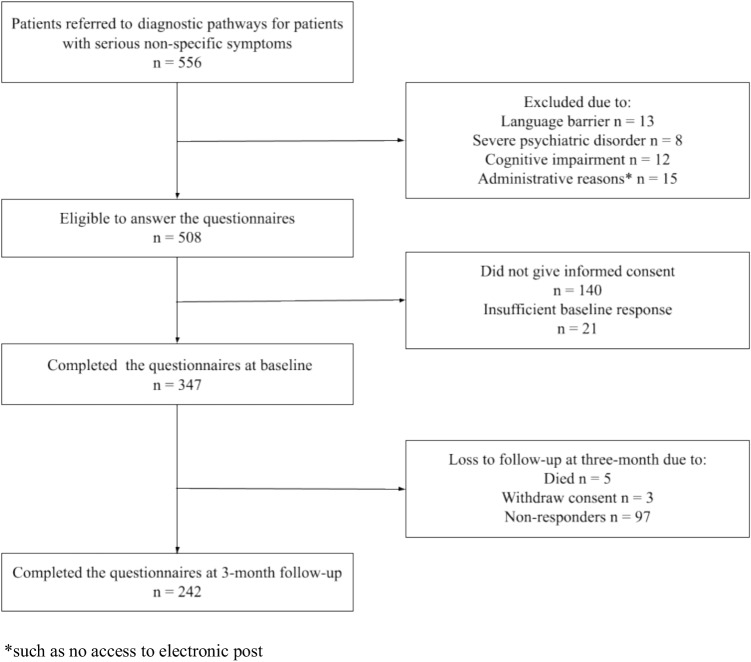
Flow of patients through the study

The patient flow is presented in Fig. 1. Patients who did not provide consent were similar in age to those who did (mean, 65 years) but included a slightly higher proportion of males (57%). Patients lost to 3-month follow-up were significantly younger (mean, 60 years) than those who completed the 3-month follow-up (mean, 68 years). No statistically significant differences in other baseline characteristics were observed. Loss to follow-up rates were 32% in the cancer group, 32% in the group with other serious diagnoses, and 29% in the with no serious diagnoses.

Table 1 outlines sociodemographic and clinical characteristics for those who completed the questionnaires at baseline.

**Table 1:** Sociodemographic and clinical characteristics at baseline

	Baseline responders *n* = 347
Sex Female, *n* (%)	174 (50)
Age Mean (SD) Range	66 (14.3) 23–90
Cohabitation status, *n* (%) Married/cohabiting Living alone Other	259 (75) 82 (24) 6 (2)
Education, *n* (%) Primary school ≤ 10 years Low ≤ 14 years Medium ≤ 17 years High > 17 years	69 (20) 177 (51) 78 (23) 23 (7)
Occupation, *n* (%) Employed/self-employed Unemployed Activity/sickness compensation Retired Other	101 (29) 6 (2) 45 (13) 191 (55) 4 (1)
Smoking habits, *n* (%) Non-smoker Former smoker Smoker	130 (38) 148 (43) 69 (20)
Drinking habits, *n* (%) ≤ 10 units per week > 10 units per week	296 (85) 51 (15)
Comorbidity (Charlson Comorbidity Index), *n* (%) None Moderate High	211 (61) 102 (29) 34 (10)

**Fig. 2:**
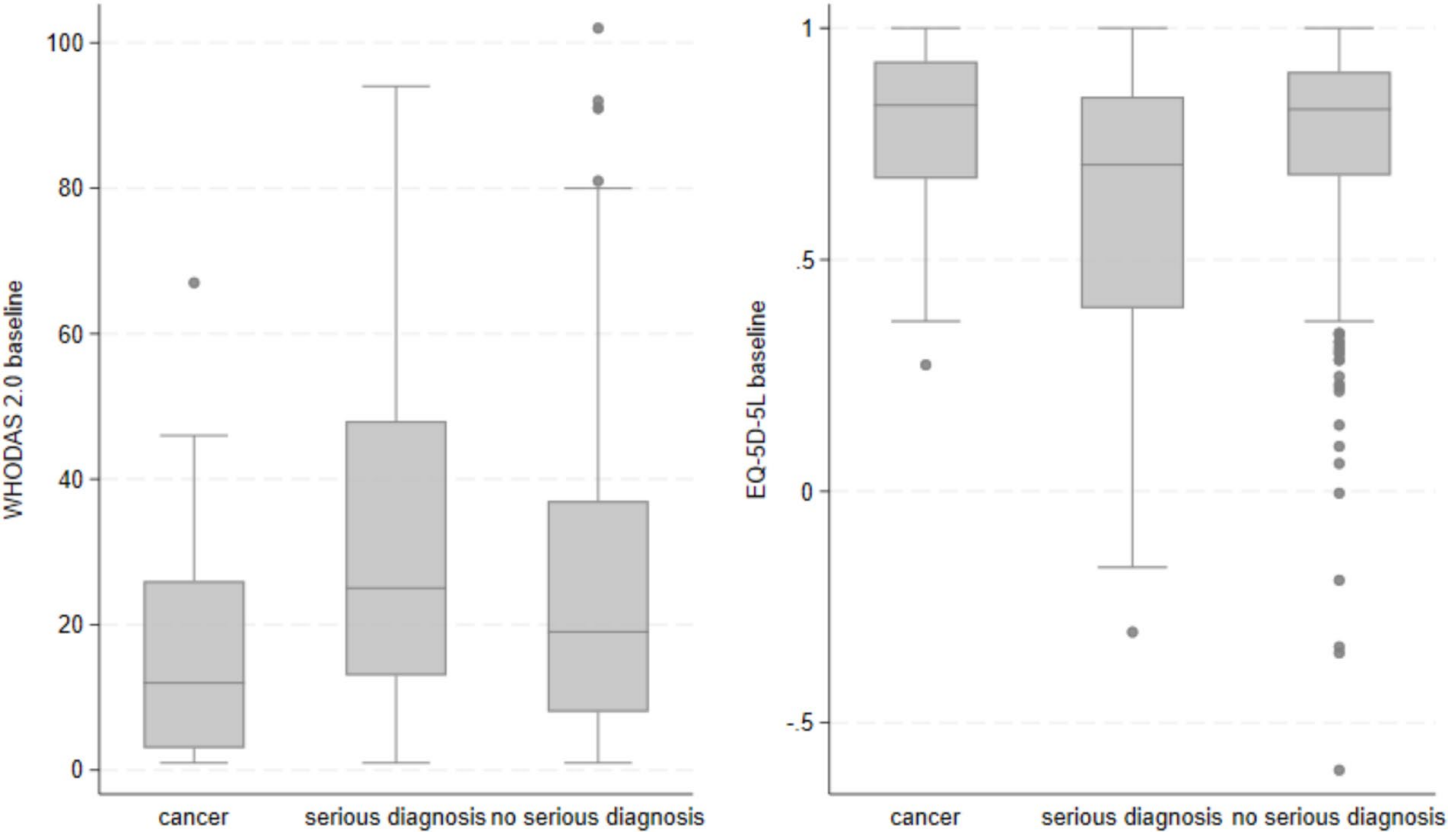
Baseline scores of WHODAS 2.0 and EQ-5D-5L for each diagnostic group

At baseline, the median WHODAS 2.0 score was 20 (IQR: 9;38), and the median EQ-5D-5L score was 0.812 (IQR: 0.586;0.888). As presented in Fig. 2, significant differences between the three diagnostic groups were seen in WHODAS 2.0 score (*p* = 0.005) and EQ-5D-5L score (*p* = 0.003). The median WHODAS 2.0 scores were: cancer 12 (IQR: 3;26), another serious diagnosis 25 (IQR: 13;48), and no serious diagnosis 19 (IQR: 8;37). The median EQ-5D-5L scores were: cancer 0.834 (IQR: 0.675;0.928), another serious diagnosis 0.705 (IQR: 0.395;0.852), and no serious diagnosis 0.825 (IQR: 0.682;0.906).

Diagnostic groups at baseline included patients with cancer (*n* = 25), another serious diagnosis (*n* = 71), and no serious diagnosis (*n* = 251). The most frequent non-malignant serious diagnoses included rheumatological, gastrointestinal, and cardiovascular conditions. A complete diagnostic overview is provided in a table (Online Resource 1). Among patients diagnosed with cancer, the most common tumor types were prostate cancer and lymphoma.

Analyses.

The distribution of WHODAS 2.0 and EQ-5D-5L scores indicates that the majority of patients reported relatively high HRQoL and low levels of functional impairment, as shown in the figure (Online Resource 2).

The mean change in WHODAS 2.0 for all three diagnostic groups combined was − 1.57 (95%CI: − 3.62;0.49); by group: cancer − 2.82 (95%CI: (− 8.60;2.96)), another serious diagnosis − 6.65 (95%CI: − 11.86; − 1.43), and no serious diagnosis − 0.07 (95%CI: − 2.40;2.27), indicating a decrease in functioning across all groups. The mean change in EQ-5D-5L for all three diagnostic groups combined was 0.061 (95%CI: 0.031;0.091); by group: cancer 0.125 (95%CI: 0.019;0.230), another serious diagnosis 0.172 (95%CI: 0.088;0.255), and no serious diagnosis 0.026 (95%CI: − 0.006;0.058), indicating improved HRQoL across all groups. However, improvement fell short of reaching a level of statistical significance in the group with no serious diagnosis.

Table 2 outlines the changes in WHODAS 2.0 and EQ-5D-5L. No significant differences in changes in WHODAS 2.0 from referral to 3-month follow-up between the diagnostic groups were observed (Table 2). In contrast, significant differences were observed in EQ-5D-5L between the diagnostic groups in the unadjusted and the adjusted analysis (Table 2). Among patients with missing follow-up on the EQ-5D-5L (*n* = 7), there were no statistically significant differences in baseline characteristics or WHODAS 2.0 scores compared to those who completed the EQ-5D-5L at the 3-month follow-up.

**Table 2:** Multiple regression analyses comparing changes in functioning and health-related quality of life scores (referral to three-month follow-up)

	Crude Estimate (95%CI)^*^	*p* value^**^	Adjusted^a^ Estimate (95%CI)^*^	*p* value^**^
Functioning (WHODAS 2.0) (0–144)				
Diagnostic groups		0.07		0.08
No serious diagnosis *n* = 177	Ref		Ref	
Another serious diagnosis *n* = 48	− 6.58 (− 8.99;3.48)		− 5.00 (− 13.05;3.05)	
Cancer diagnosis *n* = 17	− 2.76 (− 12.29; − 0.87)		− 3.42 (− 10.45; − 0.60)	
HRQoL (EQ-5D-5L) (− 0.757 to 1)				
Diagnostic groups		0.002		0.002
No serious diagnosis *n* = 173	Ref		Ref	
Another serious diagnosis *n* = 46	0.146 (0.056;0.236)		0.158 (0.067;0.249)	
Cancer diagnosis *n* = 16	0.099 (− 0.011;0.209)		0.088 (− 0.035;0.210)	

^*^95% confidence interval

^**^f test difference between groups

^a^Adjusted for sex, age, cohabitation status, education, occupation, smoking and alcohol habits, and comorbidity

Among patients with complete follow-up (*n* = 242), a significant baseline difference in EQ-5D-5L was observed between the three diagnostic groups (*p* = 0.02). No significant differences were found among other baseline characteristics (see Table 3).

**Table 3:** Sociodemographic and clinical characteristics at baseline for patients completing 3-month follow-up divided into diagnostic groups

	Three-month responders *n* = 242	Cancer diagnosis *n* = 17	Another serious diagnosis n = 48	No serious diagnosis *n* = 177
Sex, Female, *n* (%)	117 (48)	7 (41)	25 (52)	85 (48)
Age Mean (SD) Range	68 (11.8) (30–90)	67 (9.4) (41–85)	70 (12.2) (36–87)	68 (11.9) (30–90)
Cohabitation status, *n* (%) Married/cohabiting Living alone Other	180 (74) 59 (24) 3 (1)	13 (77) 4 (24)	38 (79) 10 (21)	129 (73) 45 (25) 3 (2)
Education, *n* (%) Primary school ≤ 10 years Low ≤ 14 years Medium ≤ 17 years High > 17 years	47 (19) 125 (52) 52 (22) 18 (7)	3 (18) 5 (29) 7 (41) 2 (12)	12 (25) 27 (56) 6 (13) 3 (6)	32 (18) 93 (53) 39 (22) 13 (7)
Occupation, *n* (%) Employed/self-employed Unemployed Activity/sickness compensation Retired Other	66 (27) 3 (1) 23 (10) 147 (61) 3 (1)	4 (24) 1 (6) 1 (6) 11 (65)	10 (21) 1 (2) 2 (4) 34 (71) 1 (2)	52 (29) 1 (1) 20 (11) 102 (58) 2 (1)
Smoking, *n* (%) Non-smoker Former smoker Smoker	90 (37) 108 (45) 44 (18)	4 (24) 10 (59) 3 (18)	19 (40) 20 (42) 9 (19)	67 (38) 78 (44) 32 (18)
Drinking habits, *n* (%) ≤ 10 units per week > 10 units per week	206 (85) 36 (15)	12 (71) 5 (29)	44 (92) 4 (8)	150 (85) 27 (15)
Comorbidity (Charlson Comorbidity Index) *n* (%) None Moderate High	137 (57) 77 (32) 28 (12)	10 (59) 7 (41) 0 (0)	7 (15) 30 (63) 11 (23)	98 (55) 61 (35) 18 (10)
Functioning (WHODAS 2.0) (0–144) Median, (IQR*)	18 (8;36)	12 (2;26)	23.5 (11.5;43.5)	17 (7;36)
Health-related quality of life (EQ-5D-5L) (− 0.757 to 1) Median (IQR*)	0.812 (0.616;0.901)	0.834 (0.634;0.928)	0.747 (0.429;0.864)	0.830 (0.715;0.911)

^*^Interquartile range

## Discussion

Functioning difficulties and reduced HRQoL were evident in patients referred to a cancer diagnostic pathway for serious non-specific symptoms at the time of referral. Among the diagnostic groups, those with a serious diagnosis other than cancer were the most affected compared to patients diagnosed with cancer or those without a serious diagnosis.

At the 3-month follow-up, all three diagnostic groups continued to experience functioning difficulties and reduced HRQoL. While changes in functioning from baseline to follow-up did not differ significantly between the groups, changes in HRQoL did. Patients with another serious diagnosis showed significant improvement in HRQoL at 3 months.

In Scandinavia, the WHODAS 2.0 (36 item) has reported a median score of 15.2 for the general population[29]. In contrast, patients in this study had a baseline median score of 20, indicating greater functioning difficulties compared to the general population. Patients undergoing diagnostics for possible cancer experience compromised HRQoL[30, 31], which was also observed in our study at baseline. A study reported an EQ-5D-5L average score of 0.900 among the general Danish population[32]. In contrast, our study’s population had a median baseline score of 0.812, indicating lower HRQoL compared to the general population. The result of this study shows higher level of functioning difficulties and a lower HRQoL in patients referred to a cancer diagnostic pathway than in the general population.

At baseline, the three diagnostic groups displayed varying functioning difficulties and HRQoL levels. Notably, the group with another serious diagnosis exhibited the highest degree of functioning difficulties and the lowest HRQoL, while the cancer-diagnosed group was the least affected. These differences suggest that functioning and HRQoL at referral are not solely linked to a cancer diagnosis. Varying symptom patterns in patients referred for cancer diagnostics differently affect functioning and HRQoL[9, 33].

A systematic review indicated that up to 50% of cancer patients experience disability and require assistance with daily activities[34]. However, our study did not reflect this, as the group diagnosed with cancer had the least affected functioning and HRQoL, likely due to being early in their diagnostic journey. This is supported by another review showing high HRQoL in patients with cancer in the early stages compared to patients with other diseases[35]. However, our findings may be influenced by dropout rates and the small sample size. Unlike EQ-5D-5L, WHODAS 2.0 is less commonly used and validated, both generally and in cancer populations. Furthermore, WHODAS 2.0 is a relatively lengthy questionnaire with 36 items, raising the question of whether alternative, less burdensome measurement tools should be considered. However, there is a limited choice of questionnaires that effectively measure functioning. The primary advantage of WHODAS 2.0 lies in its integration with ICD-11, its alignment with the ICF, and its use as a generic questionnaire[22].

A key strength to this study is that it is the first to investigate functioning and HRQoL within a specific population of patients with non-specific serious symptoms referred to a cancer diagnostic pathway. Despite the population’s vulnerability, we achieved a high response rate (68%). This was made possible by an additional invitation early in data collection, boosting baseline response rates from 60 to 68%. We collected a large dataset over 12 months, and the 3-month follow-up allowed for the inclusion of incident diagnosis associated with the diagnostic pathway. Another significant strength is the robust analysis, which incorporated relevant covariates. Dropout at the 3-month follow-up is a limitation. Patients lost to follow-up were significantly younger at baseline, with responders averaging 68 years (responders) and non-responders 60 years. However, this age difference was not considered clinically important. Furthermore, the relatively small number of patients diagnosed with cancer (*n* = 17) may limit the statistical power to detect differences in functioning and HRQoL changes over time in this subgroup. We do not know the patients’ subsequent course of treatment and how it may affect functioning and HRQoL. Despite these limitations, the study’s findings remain valuable for clinical practice and research, providing useful insights into patients’ functioning and HRQoL.

This study enhances our understanding of patients’ functioning and HRQoL at the time of referral to a cancer diagnostic pathway. Our results highlight the importance of systematically assessing functioning and HRQoL for all patients referred for serious non-specific symptoms rather than focusing solely on those diagnosed with cancer. Patients with cancer, other serious diagnoses, and even those without a diagnosis reported functioning difficulties and reduced HRQoL, underscoring the need for personalized care strategies that include rehabilitation needs assessment. These results support the implementation of screening tools to identify patients with rehabilitation needs, thereby enhancing rehabilitative efforts for this population. Early rehabilitation interventions can prevent or postpone the escalation of functioning difficulties and the worsening of symptoms and underlying conditions[36, 37]. Given this context, WHODAS 2.0, to be integrated into the ICD11, emerges as a promising screening tool. Its implementation as a patient-reported outcome measure could be incorporated into routine clinical practice to identify patients with unmet needs more effectively.

## Conclusion

Patients referred to a cancer diagnostic pathway for non-specific serious symptoms experience considerable negative impacts on functioning and HRQoL at the time of referral, with those diagnosed with another serious disease being the most affected. No significant differences in changes in functioning from baseline to the 3-month follow-up were observed among patients diagnosed with cancer, another serious disease, or no serious diagnosis. However, HRQoL significantly differed from baseline to 3-month follow-up among these groups. Patients with another serious diagnosis showed significantly greater improvement in HRQoL than those diagnosed with cancer or those without a serious diagnosis.

This study provides new insights into functioning and HRQoL among patients with serious non-specific symptoms referred to a cancer diagnostic pathway. This knowledge should inform the design of individualized cancer pathways, allowing additional support for those with a rehabilitation need.

## Supplementary Information

Below is the link to the electronic supplementary material.

Supplementary file1 (PDF 93 KB)

Supplementary file2 (PDF 146 KB)

## Data Availability

No datasets were generated or analysed during the current study.
