# Supplementary material for: Exploring functioning and health-related quality of life in patients referred to a diagnostic cancer pathway for non-specific serious symptoms
Source: Support Care Cancer. 2025 Aug 20;33(9):800. doi: 10.1007/s00520-025-09825-8 (PMC12367924; doi:10.1007/s00520-025-09825-8)
Supplement: Supplementary file 1 — Supplementary file1 (PDF 93 KB) [file 520_2025_9825_MOESM1_ESM.pdf]

Article title:
Exploring functioning and Health-Related Quality of Life in Patients Referred to a Diagnostic Cancer pathway for Non-Specific Serious Symptoms

Journal name: Supportive Care in Cancer

Author names:
Jannie Rhod Bloch-Nielsen, Thomas Maribo, Helene Nørgaard Kristensen, Jaana Paltamaa, Anne Mette Schmidt

Corresponding author:
Jannie Rhod Bloch-Nielsen
Medical Diagnostic Centre, University Clinic for Innovative Patient Pathways, Regional Hospital Central Jutland, Silkeborg, Denmark
Department of Physiotherapy and Occupational Therapy, Silkeborg Regional Hospital
Falkevej 1-3, 8600 Silkeborg, Denmark
Tel.: +45 24926168
E-mail: jannie.blochNielsen@midt.rm.dk

Online Resource 1:
Distribution of diagnostic categories among patients with another serious diagnosis who completed the baseline questionnaires (n = 71)

Diagnostic category	n	%
Rheumatological	24	34
Gastrointestinal	10	14
Endocrinological	6	9
Respiratory	4	6
Cardiological	12	17
Neurological	6	9
Other	9	11
Total	71	100

Categories are based on main ICD-10 diagnosis recorded three months after referral. “Other” includes infectious, haematological, nephrological, and non-specific conditions not fitting into the predefined categories.
