# Supplementary material for: Exploring functioning and health-related quality of life in patients referred to a diagnostic cancer pathway for non-specific serious symptoms
Source: Support Care Cancer. 2025 Aug 20;33(9):800. doi: 10.1007/s00520-025-09825-8 (PMC12367924; doi:10.1007/s00520-025-09825-8)
Supplement: Supplementary file 2 — Supplementary file2 (PDF 146 KB) [file 520_2025_9825_MOESM2_ESM.pdf]

Article title:
Exploring functioning and Health-Related Quality of Life in Patients Referred to a Diagnostic Cancer pathway for Non-Specific Serious Symptoms

Journal name: Supportive Care in Cancer

Author names:
Jannie Rhod Bloch-Nielsen, Thomas Maribo, Helene Nørgaard Kristensen, Jaana Paltamaa,
Anne Mette Schmidt

Corresponding author:
Jannie Rhod Bloch-Nielsen
Medical Diagnostic Centre, University Clinic for Innovative Patient Pathways, Regional Hospital Central Jutland, Silkeborg, Denmark
Department of Physiotherapy and Occupational Therapy, Silkeborg Regional Hospital
Falkevej 1-3, 8600 Silkeborg, Denmark
Tel.: *+45 24926168*
E-mail: jannie.blochNielsen@midt.rm.dk

Online Resource 2:

Agreement between baseline and three-month follow-up on WHODAS 2.0 and health-related quality of life (EQ-5D-5L)

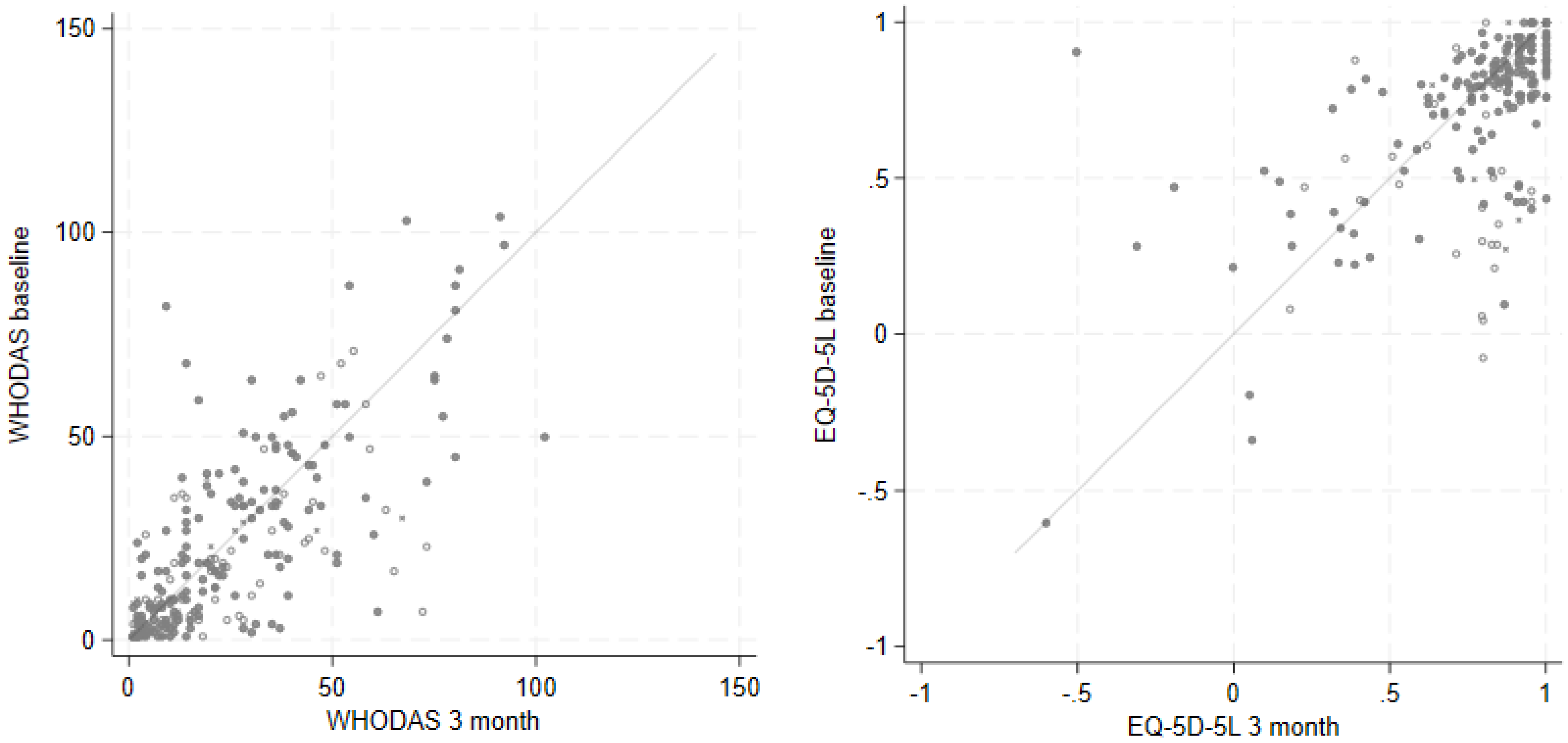

X cancer diagnosis ● another serious diagnosis, ○ no **serious** diagnosis
